# Distinct expression and prognostic values of GATA transcription factor family in human ovarian cancer

**DOI:** 10.1186/s13048-022-00974-6

**Published:** 2022-04-29

**Authors:** Quan Zhou, Huai-jie Yang, Man-zhen Zuo, Ya-ling Tao

**Affiliations:** Department of Gynecology and Obstetrics, the People’s Hospital of China Three Gorges University/the First People’s Hospital of Yichang, 2, Jie-fang Road, Yi chang, Yichang, 443000 Hubei China

**Keywords:** GATA, Ovarian cancer, Prognosis, KM plotter

## Abstract

Accumulated studies have provided controversial evidences of expression patterns and prognostic value of the *GATA* family in human ovarian cancer. In the present study, we accessed the distinct expression and prognostic roles of 7 individual members of *GATA* family in ovarian cancer (OC) patients through Oncomine analysis, CCLE analysis, Human Protein Atlas (HPA), Kaplan–Meier plotter (KM plotter) database, cBioPortal and Metascape. Our results indicated that *GATA1*, *GATA3*, *GATA4* and *TRPS1* mRNA and protein expression was significantly higher in OC than normal samples. High expression of *GATA1*, *GATA2*, and *GATA4* were significantly correlated with better overall survival (OS), while increased *GATA3* and *GATA6* expression were associated with worse prognosis in OC patients. *GATA1*, *GATA2*, *GATA3* and *GATA6* were closely related to the different pathological histology, pathological grade, clinical stage and TP53 mutation status of OC. The genetic variation and interaction of the *GATA* family may be closely related to the pathogenesis and prognosis of OC, and the regulatory network composed of *GATA* family genes and their neighboring genes are mainly involved in Notch signaling pathway, Th1 and Th2 cell differentiation and Hippo signaling pathway. Transcriptional *GATA1/2/3/4/6* could be prognostic markers and potential therapeutic target for OC patients.

**Supplementary Information:**

The online version contains supplementary material available at 10.1186/s13048-022-00974-6.

## Introduction

Ovarian cancer (OC) is the most cause of cancer-related death form of all gynecological malignancies [1, 2]. Although standard cytoreductive surgery and platinum based chemotherapy have improved overall survival and life quality, long-term survival of advanced OC patients remains poor [3]. Over 75% of patients are not early diagnosed until advanced stages, and the 5-year rate survival is less than 30%, due to the lack of specific symptoms and efficiently prognostic biomarkers [4, 5]. Therefore, further investigation on the mechanisms of OC tumorigenesis and tumor progression, and identification of potential effective and minimally prognostic markers and potential drug targets is still needed for OC patients [3].

The *GATA* protein family has been identified as one of the zinc finger DNA binding proteins that play an essential role during epithelial proliferation and development of diverse tissues [6]. Based on initial studies of their expression, *GATA1*, *GATA2*, and *GATA3* were categorized as hematopoietic *GATA* factors, while *GATA4*, *GATA5*, and *GATA6* were termed endodermal *GATA* factors [6, 7]. In biological function, *GATA1* and *GATA2* play pivotal roles in regulating cell cycle or proliferation [8]. *GATA3* is not only an important transcriptional factor for T-cell development, but it is also involved in cellular proliferation, development, and differentiation in luminal epithelial and urothelial epithelium cells [9]. *GATA4*, *GATA5* and *GATA6* are expressed predominantly in endodermand mesoderm-derived tissues [10, 11]. *GATA4* and *GATA5* tend to mark fully differentiated epithelial cells and confirmed as potential tumor suppressors [12], while *GATA6* expresses in the immature proliferating cells in the intestinal crypts and classified as potential oncogene [13]. *TRPS1* (trichorhinophalangeal syndrome-1) is a novel *GATA* transcription factor that has been found to be a critical activator of mesenchymal-to-epithelial transition (MET) during embryonic development in a number of tissues [14]. There is growing evidence that deregulation of *GATA* expression is a common occurrence in several human malignancies, and distinctive role of individual *GATA* member in tumor tumorigenesis and progression [6, 7, 15]. Such as breast [16], colon [17], lung [18], gastric [19] and pancreatic cancer [20], as well as OC [21–26]. These proteins are considered having potential value to be adopted as novel biomarkers in the detection and accurate prediction of many kinds of tumors.

Although *GATA* has been identified as a crucial transcription factors in a variety of hematogenous malignancies and solid tumors, and several GATA family members (*GATA3*, *GATA4* and *GATA6*) have been shown to be related to prognosis in OC patients [21–26]. The roles of distinct different GATA members in contribution to tumorigenesis and development of OC are still lacking. In the current study, we extended the research field to OC based on large databases, with purpose of determining the expression pattern of distinct *GATA* family members in OC.

## Material and methods

### Oncomine analysis

The individual gene mRNA expression levels of *GATA* family members (*GATA1*, *GATA2*, *GATA3*, *GATA4*, *GATA5*, *GATA6* and *TRPS1*) were determined through analysis in ONCOMINE database (www.oncomine.org), which is a publicly accessible online database with cancer microarray information to facilitate discovery from genome-wide expression analyses [27, 28]. In this study, students’-test was used to generate a *p*-value for comparison between cancer specimens and normal control datasets. The fold change was defined as 1.0, *p* value was set up at 0.05 and top 10% gene rank as threshold.

### CCLE analysis

The mRNA levels of *GATA* members in a series of cancers were analyzed by CCLE database (https://portals.broadinstitute.org/ccle/home), which is an online encyclopedia of a compilation of gene expression, chromosomal copy number and massively parallel sequencing data from 947 human cancer cell lines, to facilitate the identification of genetic, lineage, and predictors of drug sensitivity [29].

### Immunohistochemistry analysis

The Human Protein Atlas (HPA) database (www.proteinatlas.org) is an international program that has been set up to allow for a systematic exploration of the human proteome. The HPA database was used to investigate and validate the protein expression of *GATA* members in OC tissues by immunohistochemistry (Scar bar =200 μm).

### The Kaplan-Meier plotter and OncoLnc database analysis

The prognostic significance of the messenger RNA (mRNA) expression of *GATA* family genes in OC was evaluated using the Kaplan-Meier plotter (www.kmplot.com), an online database including gene expression data and clinical data [30]. In this database, all OC patients’ gene expressions and survival information were established from the Gene Expression Omnibus (*GEO*), The Cancer Genome Atlas cancer datasets (*TCGA*), and the Cancer Biomedical informatics Grid (*caBIG*) [31, 32]. Simultaneously, OncoLnc (www.oncolnc.org/) online tools to validate the correlation between the expression of each GATA family genes and the prognosis of patients with OC, which combines prognostic data from The Cancer Genome Atlas (TCGA) database with mRNA, miRNA or lncRNA expression levels. The expression and prognosis data for each gene were downloaded, and Kaplan–Meier curves were drawn using online tools. HRs, 95% CIs, and log rank value were determined and displayed on the webpage. A *p* value < 0.05 was considered to be statistically significant to reduce the false positive rate.

### cBioPortal analysis

The cBioPortal for Cancer genomics is an open access resource (http://www.cbioportal.org/), providing integrative analysis of complex cancer genomics and clinical profiles from 105 cancer studies in TCGA pipeline [33]. The frequency of GATA family gene alterations (amplification, deep deletion, missense mutations), copy-number variance (CNV) from GISTIC and mRNA expression z-scores (RNA Seq V2 RSEM) were assessed using the cBioPortal for Cancer Genomics database and TCGA. In addition, co-expression and network was calculated according to the cBioPortal’s online instruction [32].

### Functional enrichment analysis

Metascape (http://metascape.org) is a free well-maintained, user-friendly gene-list analysis tool for gene annotation and analysis resource. In this study, Metascape was used to conduct pathway and process enrichment analysis of *GATA* family members and neighboring genes. The Gene Ontology (GO) terms for the biological process (BP), cellular component (CC) and molecular function (MF) categories as well as Kyoto Encyclopedia of Genes and Genomes (*KEGG*) pathways were enriched based on Metascape online tool. Only terms with *P* value < 0.01, minimum count 3, and enrichment factor > 1.5 were concerned as significant. Molecular Complex Detection (MCODE) algorithm was further applied to identify densely connected network components.

## Results

### The mRNA expression levels of GATA family members in OC

**Fig. 1:**
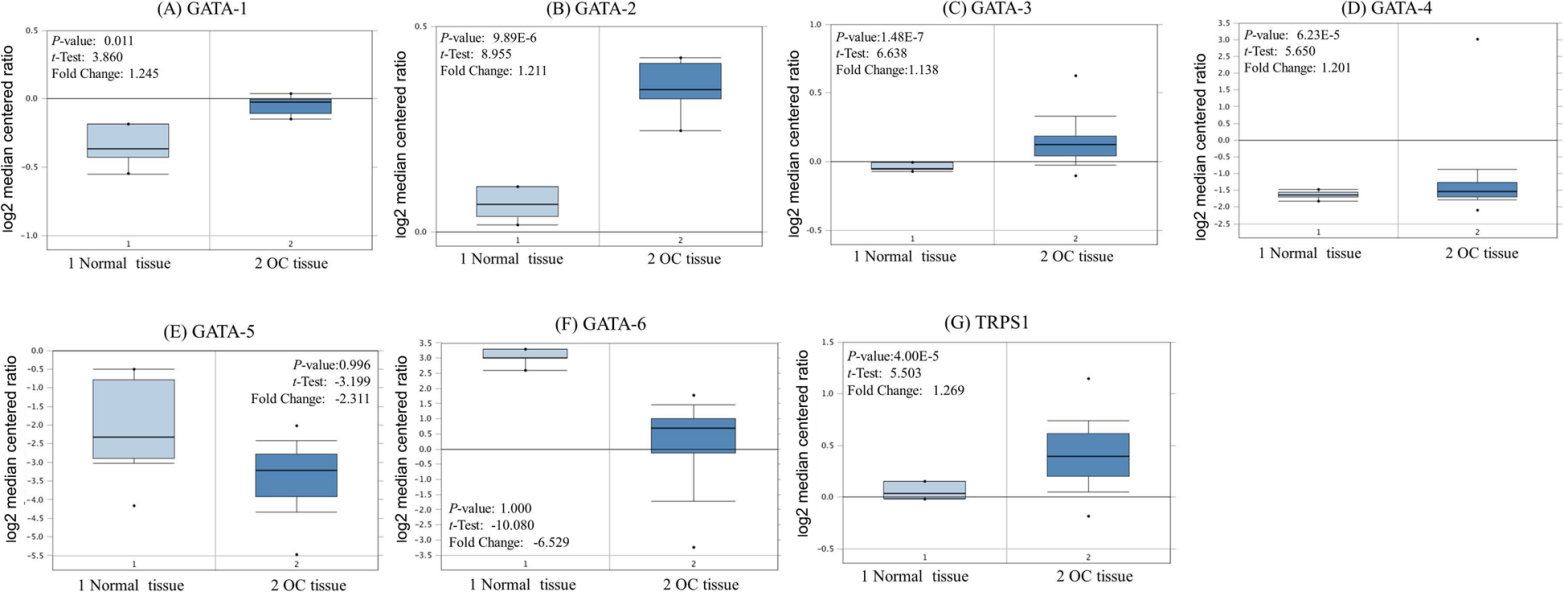
*GATA* family analysis in ovarian cancer (ONCOMINE database). **A** Comparison of *GATA1* mRNA expression (Probe IDs: 210046_at). **B** Comparison of *GATA2* mRNA expression (Probe IDs: 210358_x_at). **C** Comparison of *GATA3* mRNA expression (Probe IDs: 209604s_at). **D** Comparison of *GATA4* mRNA expression (Probe IDs: 205517_at). **E** Comparison of *GATA5* mRNA expression (Probe IDs: A_23_P132048). **F** Comparison of *GATA6* mRNA expression (Probe IDs: U66075_at). **G** Comparison of *TRPS1* mRNA expression in normal and primary OC tissues (Probe IDs: 218502_s_at)

**Fig. 2:**
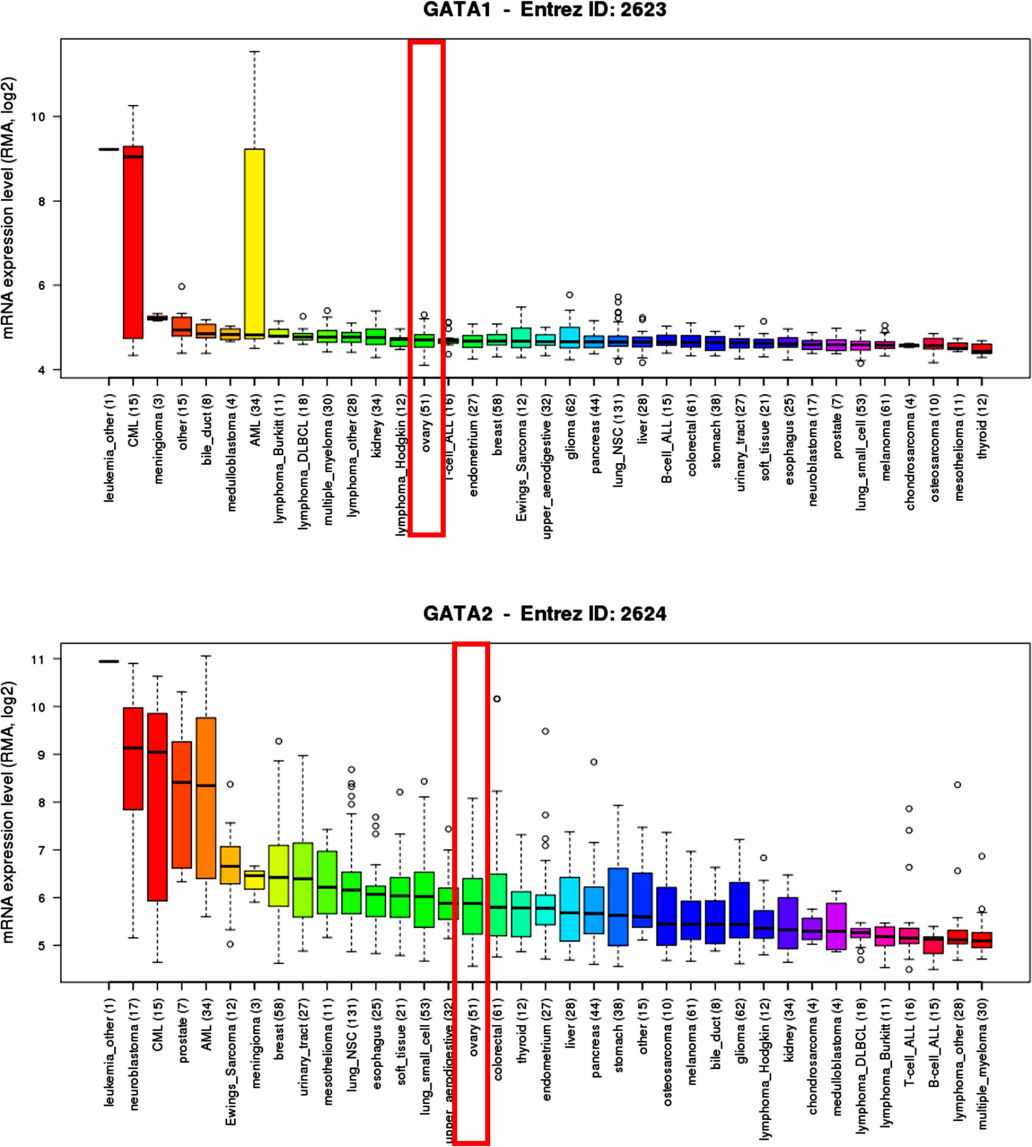
Immunohistochemistry analysis of the protein expression of GATA family members in OC patients (HPA databases). The darker the staining color, the stronger the protein expression. **A** Comparison of GATA1 protein expression in normal ovarian and OC tissues. **B** Comparison of GATA2 protein expression in normal ovarian and OC tissues. **C** Comparison of GATA3 protein expression in normal ovarian and OC tissues. **D** Comparison of GATA4 protein expression in normal ovarian and OC tissues. **E** Comparison of GATA6 protein expression in normal ovarian and OC tissues. **F** Comparison of TRPS1 protein expression in normal ovarian and OC tissues

To address the mRNA expression differences of *GATA* family between tumor and normal tissues in ovarian cancer, we performed an analysis using the Oncomine database. As shown in Fig. 1, ONCOMINE analysis revealed that *GATA1*, *GATA2*, *GATA3*, *GATA4* and *TRPS1* mRNA expression was significantly higher in OC than normal samples. *GATA1* transcripts were 1.082 fold elevated in OC samples as compared with normal tissues in a dataset with 594 samples that derived from TCGA (the Cancer Genome Atlas) database. *GATA2* was 1.211-fold elevated in OC samples as compared with normal tissues (*p* = 9.89E-6). *GATA3* was 1.138-fold elevated in OC samples as compared with normal tissues (*p* = 1.48E-7). *GATA4* was 1.201-fold elevated in OC samples as compared with normal tissues (*p* = 6.23E-5). In addition, *TRPS1* was 1.269-fold elevated in OC samples as compared with normal tissues (*p* = 4.00E-5). We chose the probe with the highest expression fold change as the Fig. 1 display when multiple probes correspond to the same GATA family member. However, no significant difference was found in the mRNA level of other *GATA* members, including *GATA5* (− 2.311 fold change, *p* = 0.996) and *GATA6* (− 2.529 fold change, *p* = 1.000) between OC samples and normal controls. CCLE analysis demonstrated that although the mRNA expression levels of GATA1 and GATA2 ranked the 14th and 16th highest in OC among different cancer cell types, the expression levels of GATA1 and GATA2 in ovarian cancer cells are generally low, (shown in green frame) (Fig. 2).

### The protein expression levels of GATA family members in OC

**Fig. 3:**
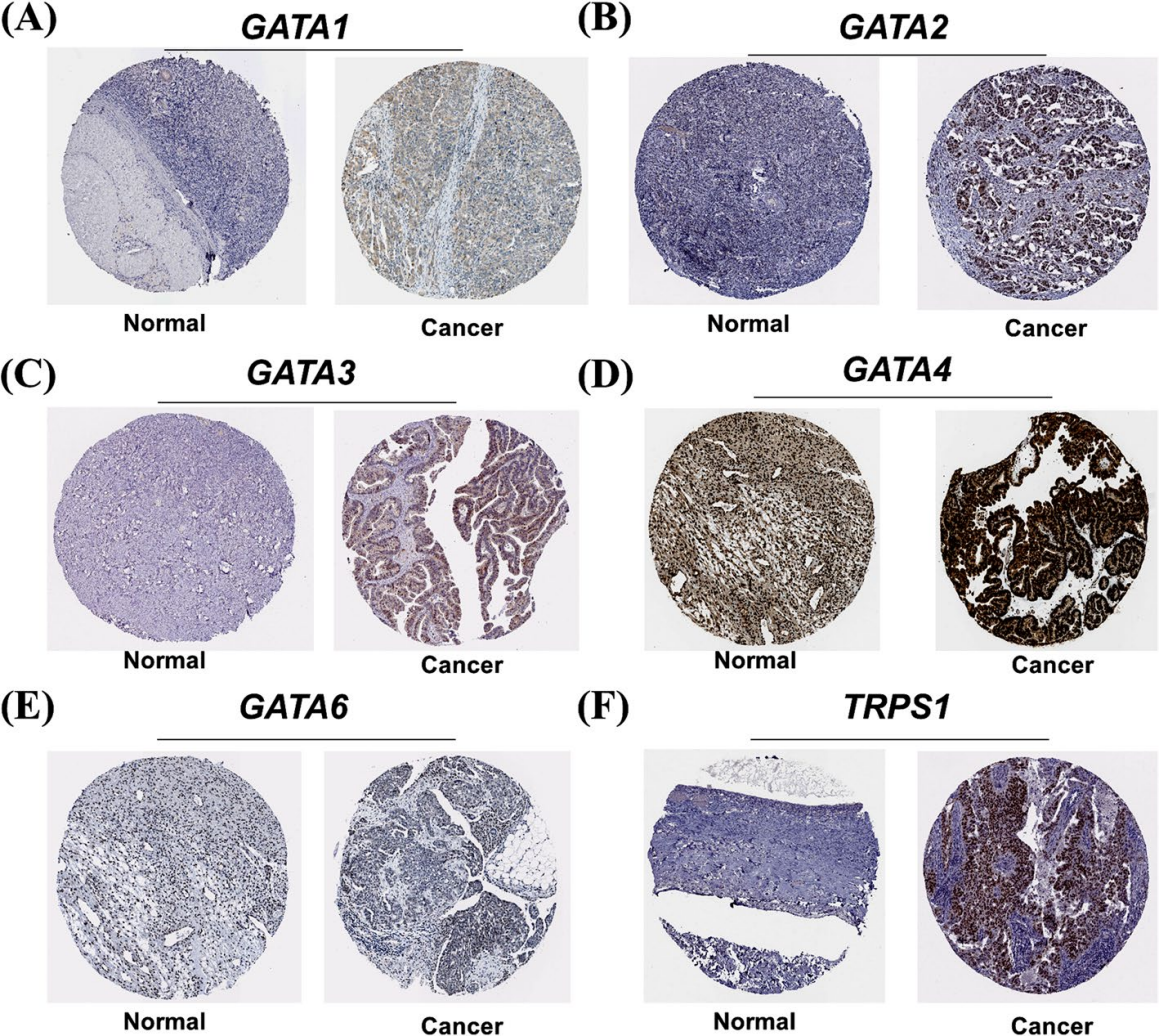
*GATA1* and *GATA2* were distinctively high expressed in ovarian cancer cell lines from CCLE analysis (CCLE database). **A** The mRNA expression levels of GATA1 ranked the 14th highest in OC among different cancer cell types. **B**The mRNA expression level of *GATA2* ranked the14th highest in a variety of cancer cell line

To further investigate and validate the protein expression level of GATA family members in OC, we performed immunohistochemistry analysis of the protein expression of GATA family members using HPA databases. In addition to *GATA5*, the protein expressions of the other 6 family members in ovarian cancer are clearly displayed in the HPA database. As shown in Fig. 3**,** we found that except for the strong staining of GATA4 in both normal and cancer tissues of the ovary, most of the GATA family members showed low expression in normal ovarian tissues, but showed moderate to high expression in OC tissues. Through the analysis of immunohistochemistry pictures, the results indicated that the protein expression of *GATA1*, *GATA2*, *GATA3*, *GATA4* and *TRPS1* also was upregulated in OC tissues compared with corresponding normal tissues.

### Prognostic values of GATA family members in OC patients

**Fig. 4:**
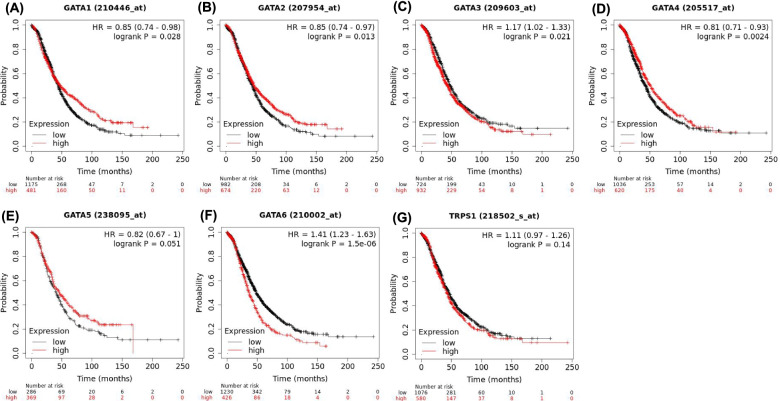
The prognostic value of mRNA level of *GATA* family members in OC patients (Kaplan-Meier plotter database). We chose the probe with the largest sample size as the target probe for further analysis when multiple probes correspond to the same GATA family member. Survival curves of (**A**) *GATA1* (Probe IDs: 210446_at), **B** *GATA2*(Probe IDs: 207954_at), **C** *GATA3*(Probe IDs: 209603_at), **D** *GATA4*(Probe IDs: 205517_at), (E)*GATA5* (Probe IDs: 238095_at), **F** *GATA6*(Probe IDs: 210002_at), **G** *TRPS1*(Probe IDs: 218502_s_at) are plotted for all patients (*n =* 1186)

We respectively examined the prognostic ability of the mRNA expression of individual GATA family members in OC patients in www.Kmplot.com. Five members were significantly associated with prognosis in OC patients (Fig. 4). We chose the probe with the largest sample size as the target probe for further analysis when multiple probes correspond to the same GATA family member. We observed that high expression of *GATA1*, *GATA2*, and *GATA4* were significantly correlated with better overall survival (OS), while increased *GATA3* and *GATA6* expression were associated with worse prognosis in OC patients. The mRNA levels of *GATA5* and *TRPS1* were not correlated with OS, although the expression of *GATA5* (hazard ratio [*HR*] = 0.82 95% confidence interval [*CI*]: 0.67–1.00, *p* = 0.0551) was modestly associated with poor survival. The prognostic values of *GATA* family members were assessed in different pathological histology subtypes of OC, including serous and endometrioid. As shown in Table 1, high mRNA expression of *GATA4* was correlated with longer OS, whereas increased *GATA6* and *TRPS1* mRNA expression were correlated with better OS in serous OC patients. In endometrioid OC, increased *GATA6* expression was associated with better prognosis. The remaining *GATA* family members were not significantly associated with prognosis in serous or endometrioid OC. Simultaneously, OncoLnc analysis demonstrated that abnormal expression of *GATA2* and *GATA4* was correlated with OS in OC patients (Logrank *P* = 0.045 and 0.042). However, the expression of other GATA family members was not statistically associated with the prognosis of patients with OC (Supplemental Information 1).

**Table 1:** Correlation of GATA gene expression level with overall survival in ovarian cancer patients with different pathological histology

GATA family	Affymetrix ID	Pathological grades	Cases	HR	95% CI	***p***-value
GATA-1	210446_at	Serous	1207	1.11	0.96-1.30	0.17
		Endometrioid	47	0.48	0.08-2.85	0.41
GATA-2	207954_at	Serous	1207	0.86	0.73-1.02	0.075
		Endometrioid	47	3.51	0.59-21.09	0.14
GATA-3	209603_at	Serous	1207	1.16	1.00-1.36	0.051
		Endometrioid	37	–	–	–
GATA-4	205517_at	Serous	1207	0.76	0.64-0.91	**0.0021**
		Endometrioid	37	6.25	0.70-56.03	0.061
GATA-5	238095_at	Serous	523	0.84	0.67-1.05	0.13
		Endometrioid	30	0.14	0.01-1.38	0.05
GATA-6	210002_at	Serous	1207	1.43	1.21-1.68	**1.5e-5**
		Endometrioid	37	5.53	0.92-33.17	**0.035**
TRPS1	218502_s_at	Serous	1104	1.44	1.25-1.67	**5.3e-7**
		Endometrioid	51	0.28	0.06-1.22	0.071

Notes: The bold values indicate that the results are statistically significant

Abbreviations: *HR* Hazard ratio, *CI* Confidence interval

We made further efforts to assess the relationship between individual GATA family members and other clinicopathological features, such as pathological grade (Table 2), clinical stage (Table 3), and TP53 status (Table 4) in OC patients. As shown in Table 2, high mRNA expression of *GATA3* was associated with worse OS in pathological grade I + II OC patients. In pathological grade III + IV OC patients, elevated mRNA expression of *GATA1*, *GATA2* and *GATA4* were associated with better OS, but high *GATA5* and *TRPS1* mRNA expression linked to poor OS. As shown in Table 3, only increased expression of *GATA3* and *GATA5* were associated with worse OS in clinical stage I patients. For clinical stage II OC patients, only high expression of *GATA4* was associated with better OS. In clinical stage III OC patients, high expression of *GATA2*, *GATA4* and *GATA5* correlated with better OS; in contrast, elevated *GATA6* expression were associated with worse OS. For clinical stage IV patients, high level of *GATA6* was associated with worse OS. Table 4 shows that the correlation between *GATA* family member expression and *TP53* status. High expression of *GATA1*, *GATA2*, *GATA3*, *GATA6* and *TRPS1* were associated with poor OS in OC patients harbouring mutated TP53. In contrast, increased *GATA2* and *GATA3* mRNA expression were linked to better prognosis, and high expression of *GATA6* was associated with linked worse OS in OC patients with wild-type TP53.

**Table 2:** Correlation of GATA gene expression level with overall survival in ovarian cancer patients with different pathological grade

GATA family	Affymetrix ID	clinical stage	Cases	HR	95% CI	***p***-value
GATA1	210446_at	I + II	135	0.68	0.30-1.54	0.36
		III + IV	1220	0.76	0.64-0.90	**0.0018**
GATA2	207954_at	I + II	135	0.52	0.22-1.20	0.12
		III + IV	1220	0.81	0.69-0.96	**0.012**
GATA3	209603_at	I + II	135	2.54	1.15-5.61	**0.017**
		III + IV	1220	0.85	0.72-1.01	0.072
GATA4	205517_at	I + II	135	0.65	0.29-1.46	0.29
		III + IV	1220	0.82	0.70-0.97	**0.018**
GATA5	238095_at	I + II	83	1.87	0.66-5.26	0.23
		III + IV	487	0.80	0.64-1.01	0.056
GATA6	217728_at	I + II	135	2.12	0.96-4.68	0.057
		III + IV	1220	1.58	1.34-1.85	**3.2e-8**
TRPS1	218502_s_at	I + II	135	1.87	0.81-4.32	0.14
		III + IV	1220	1.18	1.01-1.37	**0.035**

Notes: The bold values indicate that the results are statistically significant

Abbreviations: *HR* hazard ratio; *CI* confidence interval

**Table 3:** Correlation of GATA gene expression level with overall survival in ovarian can cer patients with different clinical stage

GATA family	Affymetrix ID	clinical stage	Cases	HR	95% CI	***p***-value
GATA-1	210446_at	I	56	0.66	0.26-1.69	0.38
		II	324	0.81	0.58-1.13	0.20
		III	1015	0.89	0.74-1.07	0.21
		IV	20	0.37	0.10-1.35	0.12
GATA-2	207954_at	I	56	0.60	0.19-1.87	0.38
		II	324	0.83	0.61-1.13	0.24
		III	1015	0.78	0.65-0.94	**0.0083**
		IV	20	0.64	0.22-1.88	0.41
GATA-3	209603_at	I	56	7.64	1.01-57.67	**0.02**
		II	324	1.36	0.98-1.89	0.066
		III	1015	1.15	0.98-1.36	0.095
		IV	20	1.65	0.64-4.27	0.29
GATA-4	205517_at	I	74	4.28	0.55-33.2	0.13
		II	61	0.34	0.11-1.03	**0.045**
		III	1044	0.81	0.68-0.96	**0.017**
		IV	176	1.30	0.82-2.05	0.26
GATA-5	238095_at	I	41	4.12	1.30-12.99	**0.0088**
		II	162	0.77	0.47-1.24	0.27
		III	392	0.75	0.58-0.96	**0.022**
		IV	18	–	–	**–**
GATA-6	210002_at	I	56	1.65	0.63-4.31	0.30
		II	324	1.24	0.89-1.72	0.20
		III	1015	1.41	1.19-1.67	**6e-05**
		IV	20	6.38	1.75-23.20	**0.0017**
TRPS1	218502_s_at	I	56	1.80	0.70-4.66	0.22
		II	324	1.33	0.98-1.80	0.068
		III	1015	1.12	0.94-1.34	0.21
		IV	20	2.79	0.77-10.05	0.10

Notes: The bold values indicate that the results are statistically significant

Abbreviations: *HR* Hazard ratio, *CI* Confidence interval

**Table 4:** Correlation of GATA gene expression level with overall survival in ovarian cancer patients with different TP53 mutation status

GATA family	Affymetrix ID	TP53 mutation	Cases	HR	95% CI	*p*-value
GATA-1	210446_at	mutated	506	1.29	1.01-1.64	**0.039**
		wild type	94	0.67	0.38-1.18	0.16
GATA-2	207954_at	mutated	506	1.37	1.09-1.72	**0.0065**
		wild type	94	0.54	0.29-0.98	**0.041**
GATA-3	209603_at	mutated	506	1.27	1.01-1.61	**0.04**
		wild type	94	0.51	0.29-0.91	**0.02**
GATA-4	205517_at	mutated	506	1.18	0.92-1.52	0.19
		wild type	94	1.37	0.79-2.37	0.27
GATA-5	238095_at	mutated	506	0.81	0.54-1.21	0.30
		wild type	19	–	–	**–**
GATA-6	210002_at	mutated	506	1.49	1.19-1.87	**0.00052**
		wild type	94	2.09	1.18-3.71	**0.0098**
TRPS1	218502_s_at	mutated	506	1.31	1.04-1.65	**0.02**
		wild type	94	0.58	0.33-1.02	0.057

Notes: The bold values indicate that the results are statistically significant

Abbreviations: *HR* Hazard ratio, *CI* Confidence interval

### Genetic alteration and neighbor gene network of GATA family members in patients with OC

**Fig. 5:**
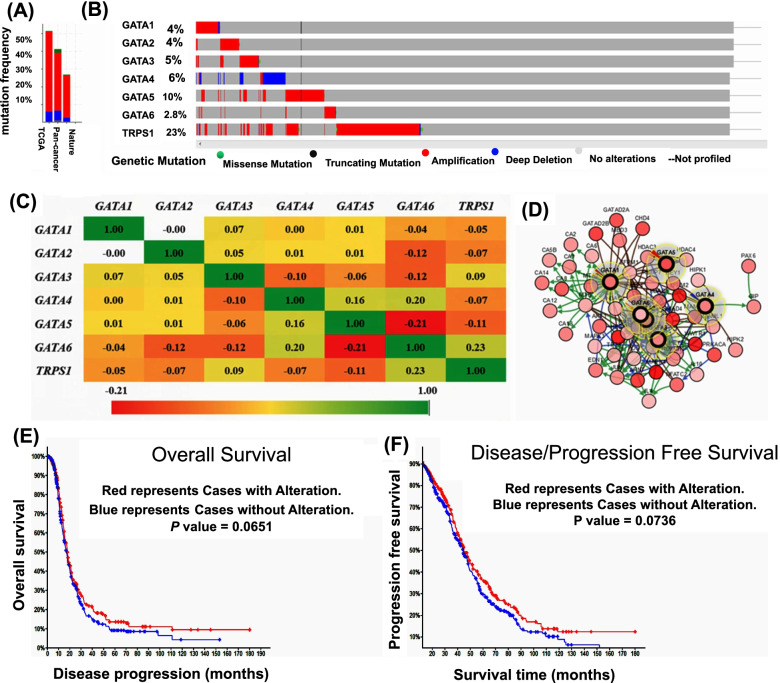
Alteration frequency and interaction analysis of *GATA* family numbers and neighbor genes network in OC patients (TCGA and cBioPortal database). **A** Summary of alteration on *GATA* family numbers. **B** OncoPrint visual summary of alteration on a query of *GATA* family numbers. **C** Pearson correlation of *GATA* family members. **D** Gene-gene interaction network among *GATA* family members in TCGA Provisional dataset, light blue represents controls state change relationship, Light green represents controls expression relationship and Brown represents the complex relationship between genes. **E** The results of Kaplan–Meier plotter and log-rank test indicated no significant difference in OS between the cases with alterations in one of the query genes and those without alterations in any query genes (*P* values, 0.0651). **F** The results of Kaplan–Meier plotter and log-rank test indicated no significant difference in DFS or PFS between the cases with alterations in one of the query genes and those without alterations in any query genes (*P* values, 0.0736)

Alteration frequency of *GATAs* mutation in OC was analyzed by using cBioPortal. A total of 1766 patients from four dataset of ovarian serous cystadenocarcinoma (TCGA Provisional), ovarian serous cystadenocarcinoma (TCGA, Nature 2011), ovarian serous cystadenocarcinoma (TCGA, PanCancer Atlas), ovarian serous cystadenocarcinoma (TCGA, Provisional) and Small Cell Carcinoma of the Ovary (MSKCC, Nat Genet 2014) were analyzed. Among this datasets analyzed, gene set/pathway is altered in 704 (40%) of queried samples for the gene sets submitted for analysis (Fig. 5A). The percentages of genetic alterations in *GATA family members* for OC varied from 4 to 23% for individual genes based on TCGA Provisional dataset (*GATA1*, 4%; *GATA2,*4%; *GATA3*,5%; *GATA4*,6%; *GATA5*,10%; *GATA6*,2.8% and *TRPS1*,23%) **(**Fig. 5B**)**. Pearson correlation analysis was conducted using expression data (*RNA Seq V2 RSEM*) of *GATA* family members collected from the cBioPortal online tool for OC. The results indicated that there is a significant positive correlation among *GATA2* with *GATA4* and *GATA5*. However, *GATA1* with *GATA2* and *GATA6* had a significant negative correlation (Fig. 5C). We then constructed the network for *GATA* and the 50 most frequently altered neighbor genes using the cBioPortal. The results showed that *AKT1*, *ARNT*, *CA13*, *CA14*, *CA2*, *CA3*, *CA4*, *CA5B*, *CA6*, *CA7*, *CA8*, *CHD4*, *CREBBP*, *EDN1*, *EP300*, *GATA1*, *GATAD2A*, *GATAD2B*, *GIP*, *HDAC1*, *HDAC2*, *HDAC3*, *HDAC4*, *HES1*, *HEY1*, *HEY2*, *HIPK1*, *HIPK2*, *IL10*, *ISL1*, *JUN*, *MAML1*, *MAML2*, *MAPK1*, *MAPK3*, *MBD3*, *MTA2*, *MYB*, *NFATC2*, *NOTCH1*, *PAX6*, *PRKACA*, *RBBP4*, *RBBP7*, *RBPJ*, *SMAD3*, *SMAD4*, *TP73*, *WWTR1*, *ZFPM1* and *ZFPM2* were closely associated with *GATA* alterations and functions (Fig. 5D). The results of Kaplan–Meier plotter and log-rank test indicated no significant difference in OS and disease-free survival (DFS) or progression-free survival (PFS) between the cases with alterations in one of the query genes and those without alterations in any query genes (*P* values, 0.0651 and 0.0736 respectively; Fig. 5E and F).

### Functions enrichment analysis of GATA family members in patients with OC

**Fig. 6:**
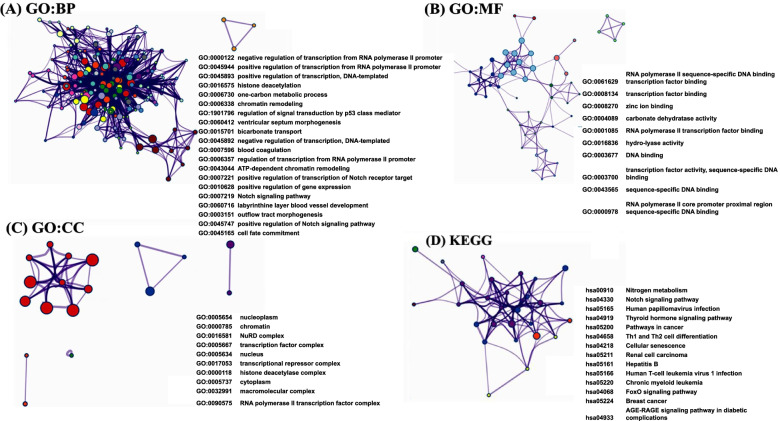
Functions enrichment analysis of *GATA* family members and their neighboring genes in patients with OC (Metascape database). The Gene Ontology (GO) terms for the (**A**) biological process (BP), (**B**) cellular component (CC), (**C**) molecular function (MF) and (**D**) Kyoto Encyclopedia of Genes and Genomes (KEGG) pathways were enriched based on Metascape online tool

The functions of *GATA* family members and their neighboring genes were predicted by analyzing gene ontology (GO) and Kyoto Encyclopedia of Genes and Genomes (KEGG) in Metascape. As shown in Fig. 6A-D and Table 5, the GO enrichment items were classified into three functional groups: biological process group, molecular function group, and cellular component group. The *GATA* family members and their neighboring genes were mainly enrichment in the heart development, embryonic organ development, regulation of binding, response to wounding, endocrine system development, regulation of Notch signaling pathway, muscle cell differentiation, regulation of hemopoiesis, regulation of stem cell differentiation, cardiac muscle hypertrophy, cytokine production, animal organ formation, muscle cell development, cellular response to hormone stimulus and response to heat; The molecular functions that these genes were mainly expressed in transcription regulatory region sequence-specific DNA binding, transcription factor binding and carbonate dehydratase activity; The cellular components that these genes were involve in the transcriptional repressor complex and transcription factor complex. The top 9 KEGG pathways for *GATA* family members and their neighboring genes are shown in Fig. 6D and Table 5. Among these pathways, the Notch signaling pathway, Th1 and Th2 cell differentiation and Hippo signaling pathway were found to relate to multiple tumor development, and it be involved in OC tumorigenesis and pathogenesis.

**Table 5:** Functions enrichment analysis of GATA family members in ovarian cancer patients

GO	Category	Description	Count	%	Log10(***P***)	Log10(***q***)
GO:0000976	GO Molecular Functions	transcription regulatory region sequence-specific DNA binding	31	54.39	−30.16	−25.82
GO:0008134	GO Molecular Functions	transcription factor binding	29	50.88	−29.79	−25.75
GO:0017053	GO Cellular Components	transcriptional repressor complex	16	28.07	−25.93	−22.49
GO:0007507	GO Biological Processes	heart development	24	42.11	−23.46	−20.07
GO:0048568	GO Biological Processes	embryonic organ development	22	38.60	−23.18	−19.84
GO:0004089	GO Molecular Functions	carbonate dehydratase activity	9	15.79	−19.73	−16.59
hsa00910	KEGG Pathway	Nitrogen metabolism	9	15.79	−19.40	−16.71
GO:0051098	GO Biological Processes	regulation of binding	18	31.58	−18.58	−15.58
GO:0009611	GO Biological Processes	response to wounding	21	36.84	−17.75	−14.83
GO:0035270	GO Biological Processes	endocrine system development	12	21.05	−15.67	−12.95
GO:0008593	GO Biological Processes	regulation of Notch signaling pathway	11	19.30	−15.18	−12.50
hsa04330	KEGG Pathway	Notch signaling pathway	9	15.79	−14.59	−12.20
GO:0042692	GO Biological Processes	muscle cell differentiation	15	26.32	−13.90	−11.34
GO:1903706	GO Biological Processes	regulation of hemopoiesis	15	26.32	−13.32	−10.81
GO:2000736	GO Biological Processes	regulation of stem cell differentiation	9	15.79	−13.14	−10.68
GO:0003300	GO Biological Processes	cardiac muscle hypertrophy	10	17.54	−13.13	−10.67
GO:0001816	GO Biological Processes	cytokine production	17	29.82	−12.30	−9.95
hsa04658	KEGG Pathway	Th1 and Th2 cell differentiation	9	15.79	−11.91	−9.92
GO:0005667	GO Cellular Components	transcription factor complex	13	22.81	−11.56	− 9.28
hsa05169	KEGG Pathway	Epstein-Barr virus infection	10	17.54	−10.21	−8.29
hsa05161	KEGG Pathway	Hepatitis B	9	15.79	−10.14	−8.29
GO:0048645	GO Biological Processes	animal organ formation	7	12.28	−9.74	−7.59
GO:0055001	GO Biological Processes	muscle cell development	9	15.79	−9.43	−7.31
GO:0032870	GO Biological Processes	cellular response to hormone stimulus	14	24.56	−9.04	−6.98
GO:0009408	GO Biological Processes	response to heat	8	14.04	−8.72	−6.68
hsa05321	KEGG Pathway	Inflammatory bowel disease (IBD)	4	7.02	−4.71	−3.66
hsa04390	KEGG Pathway	Hippo signaling pathway	4	7.02	−3.25	−2.40
hsa05031	KEGG Pathway	Amphetamine addiction	3	5.26	−3.20	−2.37
hsa05418	KEGG Pathway	Fluid shear stress and atherosclerosis	3	5.26	−2.29	−1.57

Notes: The bold values indicate that the results are statistically significant

Abbreviations: *GO* Gene Ontology, *KEGG* Kyoto Encyclopedia of Genes and Genomes

**Fig. 7:**
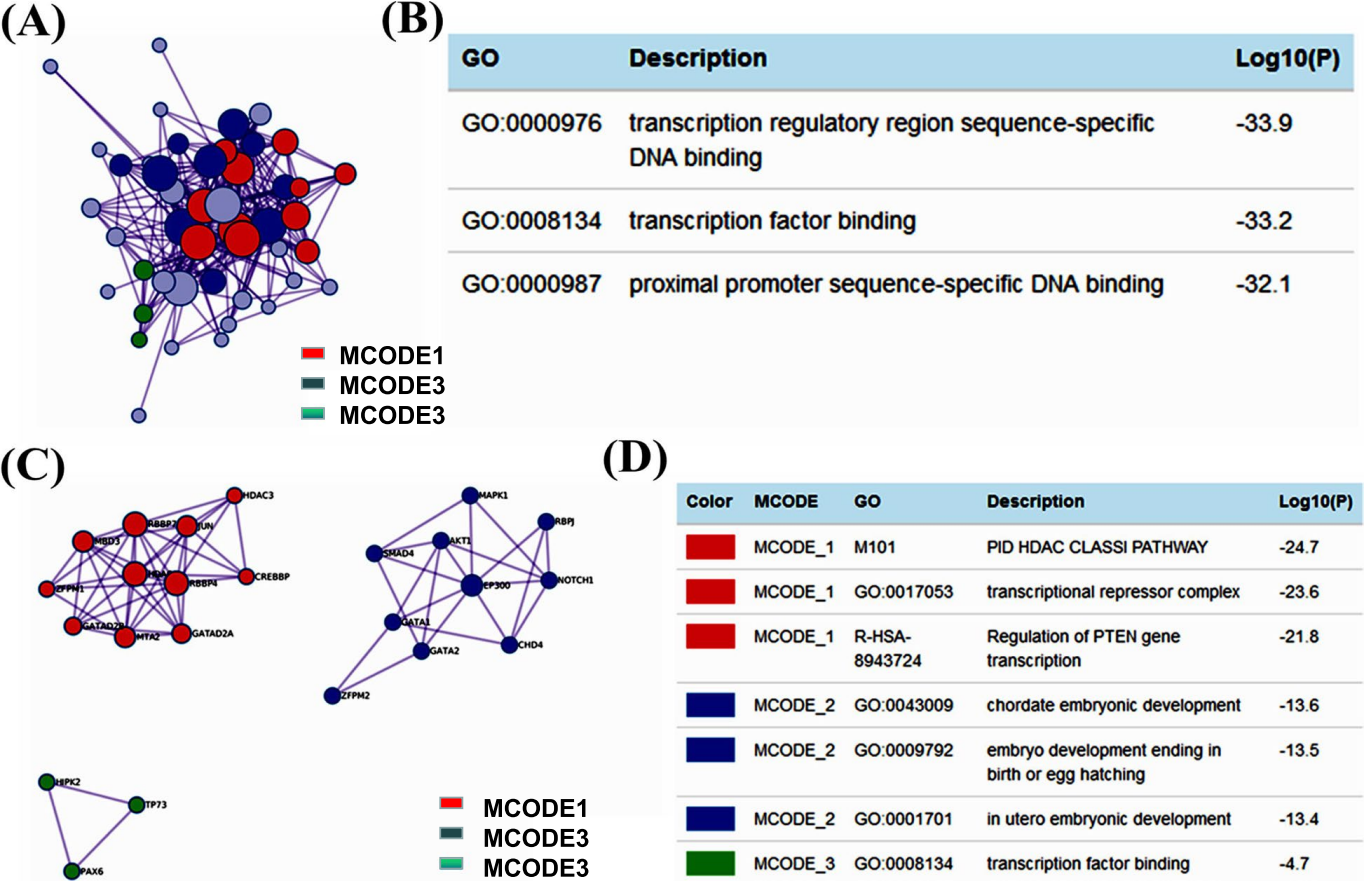
Protein-protein interaction network and MCODE components among *GATA* family members and their neighboring genes (Metascape database). **A** Protein-protein interaction (PPI) network. **B** GO Function enrichment analysis of PPI network. **C** Three most significant MCODE components form PPI network. **D** Function enrichment analysis of three MCODE components independently among GATA family members and their neighboring genes

In addition, to better understand the relationship between *GATA* family members and OC, we performed a Metascape protein-protein interaction (PPI) enrichment analysis and module analysis of the PPI network. The PPI network and MCODE components identified in the gene lists and shown in Fig. 7A-D. The PPI network were significantly associated with heart development, embryonic organ development and chordate embryonic development, while in three significant modules, GO term enrichment analysis of biological processes showed that the genes in these modules were mainly associated with ATP-dependent chromatin remodeling, histone deacetylation, protein deacetylation, chordate embryonic development, embryo development ending in birth or egg hatching and in utero embryonic development.

## Discussion

*GATA* family has been widely recognized as pivotal transcription factors in the development and differentiation of various cell types in vertebrates. Increasing evidence has shown that altered expression of *GATA* factors plays an important role in dedifferentiation of ovarian carcinogenesis. However, the exact role of *GATA* expression in OC is still controversial. In the current study, we comprehensively examined the expression patterns and prognosis analyses of individual *GATA* family members in OC using the Oncomine database, the CCLE database, the KM plotter, cBioPortal and Metascape. Our analysis suggested that, among the members of the *GATA* family, *GATA1*, *GATA3*, *GATA4* and *TRPS1* mRNA expression was significantly higher in OC than normal samples. The mRNA expression level of *GATA1* and *GATA2* in OC listed the moderate highest among all cancer types using the CCLE analysis. More importantly, survival analysis indicated that high expression of *GATA1*, *GATA2*, and *GATA4* were significantly correlated with better OS, while increased *GATA3* and *GATA6* expression were associated with worse prognosis in OC patients. We further assessed the prognostic value of *GATA* in different pathological grades, clinical stages and *TP53* mutation status of OC patients. The results showed that *GATA1*, *GATA2*, *GATA3* and *GATA6* were closely related to the different clinicopathological features and treatment of OC. Then, we tried to systematically explore the genetic alteration, correlation and potential functions of *GATA* family numbers in OC. Our findings confirmed that the genetic variation and interaction of the *GATA* family may be closely related to the pathogenesis and prognosis of OC, and the regulatory network composed of GATA family genes and their neighboring genes are mainly involved in Notch signalling pathway, Th1 and Th2 cell differentiation and Hippo signalling pathway.

*GATA1*, the first recognised member of the *GATA* family, is essential for erythropoiesis, megakaryocyte maturation, and eosinophil production [34]. The observations in human patients confirmed the critical role for *GATA1* in erythroid and megakaryocytes development, and *GATA1* mutations may be closely related to two neoplastic diseases: transient myeloproliferative disorder and acute megakaryoblastic leukemia [35]. However, its role in solid tumour has not yet been fully elucidated [36]. Our results demonstrated that increased expression of *GATA1* was correlated with significantly better OS for all OC patients, but not in serous or endometrioid subtype patients. This may be due to the small sample size of these two subtypes. Two previous studies found that *GATA1* and its phosphorylation may play an important role in the metastasis of breast cancer, and *GATA1* can be used as an independent prognostic marker for breast cancer [37, 38]. Unfortunately, as far as I know, no molecular biology studies have directly explored the prognostic value of *GATA1* for OC. This study further shows that high expression of *GATA1* indicated a better OS for OC patients with high stage (III + IV). Furthermore, the 11% of genetic alterations in *GATA1* for OC based on TCGA Provisional dataset, and *GATA1* with *GATA2* and *GATA6* had a significant negative correlation through Pearson correlation analysis. Due to the lack of relevant research, the conclusion of our study on *GATA1* needs to be further confirmed.

*GATA2* is identified as a critical regulator of growth, differentiation and survival of hematopoietic stem cells [39, 40]. Increasing evidence has shown that *GATA2* expression is correlated with hematologic pathophysiologies and the proliferation and progression of solid tumors [40]. Upregulated *GATA2* expression has been implicated in several tumour types, such as breast cancer [41], colorectal cancer [42] and liver cancer [43]. Moreover, recent studies confirmed that *GATA2* overexpression in prostate cancer increases cellular motility and invasiveness, proliferation, tumorigenicity, and resistance to standard therapies [40]. In our study, high expression of *GATA2* was significantly associated with better OS, especially in pathological grade III + IV OC patients. In addition, increased *GATA2* expression was linked to better prognosis in OC patients with wild-type *TP53* in our analysis.

*GATA3* is a “master regulator” in both mouse and human development that plays a critical role in multi-organ development and regulates tissue specific cellular differentiation [44]. It is reported to be abnormal expressed in breast and urothelial carcinomas and, hence, has been used as a marker and extensively investigated in these cancers [44, 45]. Recent evidence suggests that *GATA3* as a strong and independent predictor of clinical outcome in human luminal breast cancer [16, 46]. Lower *GATA3* expression is strongly associated with higher histologic grade, poor differentiation, positive lymph nodes, ER − and progesterone receptor (*PR*) negative status, *HER2/neu* overexpression and all other indicators of poor prognosis [46]. The presumed role of *GATA3* in the pathogenesis of OC, however, still remains unclear [47]. Our analysis showed that overexpression of *GATA3* was associated with worse prognosis in OC patients, especially in early clinical stages, patients undergoing optimal surgery and two pathological types of OC.

*GATA4*, *GATA5*, and *GATA6* are expressed predominantly in endoderm and mesoderm-derived tissues [10]. As to the intestinal cell types of expression, it has been suggested that *GATA4* and *GATA5* tend to mark fully differentiated epithelial cells [48], while *GATA6* is expressed in the immature proliferating cells in the intestinal crypts [49]. Thus, *GATA4* and *GATA5* is currently considered potential tumour suppressors, however, *GATA6* can be used as a potential oncogene [6]. Altered expression of *GATA4*, *GATA5*, and *GATA6* are associated with abroad range of tumours emerging from the gastrointestinal tract [50], lungs [51] and brain [52]. Moreover, some studies reported that methylation in the *GATA4* and *GATA6* promoter region could play an important role in ovarian carcinogenesis, elevated *GATA4* and lower *GATA6* mRNA levels are associated with better prognosis in ovarian tumours [21, 22, 25]. We found a similar result, with high *GATA4* expression being related to better prognosis in OC patients, and increased *GATA6* expression were associated with worse prognosis in OC patients. Although several studies have shown that the expression and methylation states of *GATA5* may be involved in ovarian carcinogenesis. The biologic role and the prognostic effect of *GATA5* in OC patients are still poorly understood. Our study suggests that there is a significant positive correlation among *GATA2* with *GATA4* and *GATA5*, the 10% of genetic alterations in *GATA5* for OC based on TCGA dataset. Regrettably, the expression level of *GATA5* is not related to the OS of OC.

## Conclusion

In conclusion, the members of the *GATA* family, *GATA1*, *GATA3*, *GATA4* and *TRPS1* mRNA expression was significantly higher in OC than normal samples. High expression of *GATA1*, *GATA2*, and *GATA4* were significantly correlated with better OS, while increased *GATA3* and *GATA6* expression were associated with worse prognosis in OC patients. The genetic variation and interaction of the *GATA* family may be closely related to the pathogenesis and prognosis of OC, and the regulatory network composed of *GATA* family genes and their neighboring genes are mainly involved in Notch signalling pathway, *Th1* and Th2 cell differentiation and Hippo signalling pathway. Transcriptional GATA1/2/3/4/6 could be prognostic markers and potential therapeutic target for OC patients.

## Supplementary Information

**Additional file 1: Fig. S1.** The prognostic value of mRNA level of GATA family members in OC patients (OncoLnc online tool). There is no information in the OncoLnc database that correlates GATA1 expression with ovarian cancer prognosis. (A) GATA2 (Logrank *p* = 0.045), (B)GATA3 (Logrank *p*-value = 0.467), (C)GATA4(Logrank *p*-value = 0.042), (D)GATA5(Logrank *p*-value = 0.575), (E)GATA6 (Logrank *p*-value = 0.973), (F)TRPS1(Logrank *p*-value = 0.575) are plotted for all patients (*n* = 273)

## Data Availability

The data used in this study were obtained from published reports, and there is no need to provide additional statement of permission/consent for these databases. All data generated or analysed during this study are included in this published article.

## References

[1] Siegel RL, Miller KD, Jemal A (2017). Cancer statistics, 2017. CA Cancer J Clin.

[2] Torre LA, Bray F, Siegel RL, Ferlay J, Lortet-Tieulent J, Jemal A (2015). Global cancer statistics, 2012. CA Cancer J Clin.

[3] Jin J (2018). Screening for Ovarian Cancer Jama.

[4] Force USPST, Grossman DC, Curry SJ, Owens DK, Barry MJ, Davidson KW (2018). Screening for ovarian Cancer: US preventive services task Force recommendation statement. Jama.

[5] Kim SJ, Rosen B, Fan I, Ivanova A, McLaughlin JR, Risch H (2017). Epidemiologic factors that predict long-term survival following a diagnosis of epithelial ovarian cancer. Br J Cancer.

[6] Zheng R, Blobel GA (2010). GATA transcription factors and Cancer. Genes & cancer.

[7] Lentjes MH, Niessen HE, Akiyama Y, de Bruine AP, Melotte V, van Engeland M (2016). The emerging role of GATA transcription factors in development and disease. Expert Rev Mol Med.

[8] Ohneda K, Yamamoto M (2002). Roles of hematopoietic transcription factors GATA-1 and GATA-2 in the development of red blood cell lineage. Acta Haematol.

[9] Fang SH, Chen Y, Weigel RJ (2009). GATA-3 as a marker of hormone response in breast cancer. J Surg Res.

[10] Divine JK, Staloch LJ, Haveri H, Jacobsen CM, Wilson DB, Heikinheimo M (2004). GATA-4, GATA-5, and GATA-6 activate the rat liver fatty acid binding protein gene in concert with HNF-1alpha. Am J Physiol Gastrointest Liver Physiol.

[11] Vuorenoja S, Rivero-Muller A, Kiiveri S, Bielinska M, Heikinheimo M, Wilson DB (2007). Adrenocortical tumorigenesis, luteinizing hormone receptor and transcription factors GATA-4 and GATA-6. Mol Cell Endocrinol.

[12] Akiyama Y, Watkins N, Suzuki H, Jair KW, van Engeland M, Esteller M (2003). GATA-4 and GATA-5 transcription factor genes and potential downstream antitumor target genes are epigenetically silenced in colorectal and gastric cancer. Mol Cell Biol.

[13] Maeda M, Ohashi K, Ohashi-Kobayashi A (2005). Further extension of mammalian GATA-6. Develop Growth Differ.

[14] Gai Z, Gui T, Muragaki Y (2011). The function of TRPS1 in the development and differentiation of bone, kidney, and hair follicles. Histol Histopathol.

[15] Fujiwara T (2017). GATA transcription factors: basic principles and related human disorders. Tohoku J Exp Med.

[16] Guo Y, Yu P, Liu Z, Maimaiti Y, Chen C, Zhang Y (2017). Prognostic and clinicopathological value of GATA binding protein 3 in breast cancer: a systematic review and meta-analysis. PLoS One.

[17] Shureiqi I, Zuo X, Broaddus R, Wu Y, Guan B, Morris JS (2007). The transcription factor GATA-6 is overexpressed in vivo and contributes to silencing 15-LOX-1 in vitro in human colon cancer. FASEB J.

[18] Hashiguchi T, Miyoshi H, Nakashima K, Yokoyama S, Matsumoto R, Murakami D (2017). Prognostic impact of GATA binding protein-3 expression in primary lung adenocarcinoma. Hum Pathol.

[19] Wen XZ, Akiyama Y, Pan KF, Liu ZJ, Lu ZM, Zhou J (2010). Methylation of GATA-4 and GATA-5 and development of sporadic gastric carcinomas. World J Gastroenterol.

[20] Fu B, Luo M, Lakkur S, Lucito R, Iacobuzio-Donahue CA (2008). Frequent genomic copy number gain and overexpression of GATA-6 in pancreatic carcinoma. Cancer Biol Ther.

[21] Kyronlahti A, Ramo M, Tamminen M, Unkila-Kallio L, Butzow R, Leminen A (2008). GATA-4 regulates Bcl-2 expression in ovarian granulosa cell tumors. Endocrinology..

[22] McEachin MD, Xu XX, Santoianni RA, Lawson D, Cotsonis G, Cohen C (2008). GATA-4 and GATA-6 expression in human ovarian surface epithelial carcinoma. Applied immunohistochemistry & molecular morphology : AIMM.

[23] Caslini C, Capo-chichi CD, Roland IH, Nicolas E, Yeung AT, Xu XX (2006). Histone modifications silence the GATA transcription factor genes in ovarian cancer. Oncogene..

[24] Wakana K, Akiyama Y, Aso T, Yuasa Y (2006). Involvement of GATA-4/−5 transcription factors in ovarian carcinogenesis. Cancer Lett.

[25] Capo-chichi CD, Roland IH, Vanderveer L, Bao R, Yamagata T, Hirai H (2003). Anomalous expression of epithelial differentiation-determining GATA factors in ovarian tumorigenesis. Cancer Res.

[26] Laitinen MP, Anttonen M, Ketola I, Wilson DB, Ritvos O, Butzow R (2000). Transcription factors GATA-4 and GATA-6 and a GATA family cofactor, FOG-2, are expressed in human ovary and sex cord-derived ovarian tumors. J Clin Endocrinol Metab.

[27] Rhodes DR, Yu J, Shanker K, Deshpande N, Varambally R, Ghosh D (2004). ONCOMINE: a cancer microarray database and integrated data-mining platform. Neoplasia..

[28] Rhodes DR, Kalyana-Sundaram S, Mahavisno V, Varambally R, Yu J, Briggs BB (2007). Oncomine 3.0: genes, pathways, and networks in a collection of 18,000 cancer gene expression profiles. Neoplasia..

[29] Barretina J, Caponigro G, Stransky N, Venkatesan K, Margolin AA, Kim S (2012). The Cancer cell line encyclopedia enables predictive modelling of anticancer drug sensitivity. Nature..

[30] Gyorffy B, Lanczky A, Eklund AC, Denkert C, Budczies J, Li Q (2010). An online survival analysis tool to rapidly assess the effect of 22,277 genes on breast cancer prognosis using microarray data of 1,809 patients. Breast Cancer Res Treat.

[31] Gyorffy B, Surowiak P, Budczies J, Lanczky A (2013). Online survival analysis software to assess the prognostic value of biomarkers using transcriptomic data in non-small-cell lung cancer. PLoS One.

[32] Gyorffy B, Lanczky A, Szallasi Z (2012). Implementing an online tool for genome-wide validation of survival-associated biomarkers in ovarian-cancer using microarray data from 1287 patients. Endocr Relat Cancer.

[33] Cerami E, Gao J, Dogrusoz U, Gross BE, Sumer SO, Aksoy BA (2012). The cBio cancer genomics portal: an open platform for exploring multidimensional cancer genomics data. Cancer discovery.

[34] Lowry JA, Mackay JP (2006). GATA-1: one protein, many partners. Int J Biochem Cell Biol.

[35] Migliaccio AR, Rana RA, Vannucchi AM, Manzoli FA (2005). Role of GATA-1 in normal and neoplastic hemopoiesis. Ann N Y Acad Sci.

[36] Morceau F, Schnekenburger M, Dicato M, Diederich M (2004). GATA-1: friends, brothers, and coworkers. Ann N Y Acad Sci.

[37] Zhang Y, Liu J, Lin J, Zhou L, Song Y, Wei B (2016). The transcription factor GATA1 and the histone methyltransferase SET7 interact to promote VEGF-mediated angiogenesis and tumor growth and predict clinical outcome of breast cancer. Oncotarget..

[38] Li Y, Ke Q, Shao Y, Zhu G, Li Y, Geng N (2015). GATA1 induces epithelial-mesenchymal transition in breast cancer cells through PAK5 oncogenic signaling. Oncotarget..

[39] Rodrigues NP, Tipping AJ, Wang Z, Enver T (2012). GATA-2 mediated regulation of normal hematopoietic stem/progenitor cell function, myelodysplasia and myeloid leukemia. Int J Biochem Cell Biol.

[40] Rodriguez-Bravo V, Carceles-Cordon M, Hoshida Y, Cordon-Cardo C, Galsky MD, Domingo-Domenech J (2017). The role of GATA2 in lethal prostate cancer aggressiveness. Nature reviews Urology.

[41] Wang Y, He X, Ngeow J, Eng C (2012). GATA2 negatively regulates PTEN by preventing nuclear translocation of androgen receptor and by androgen-independent suppression of PTEN transcription in breast cancer. Hum Mol Genet.

[42] Liu X, Jiang B, Wang A, Di J, Wang Z, Chen L (2015). GATA2 rs2335052 polymorphism predicts the survival of patients with colorectal Cancer. PLoS One.

[43] Hamadou WS, Mani R, Besbes S, Bourdon V, Youssef YB, Eisinger F (2017). GATA2 gene analysis in several forms of hematological malignancies including familial aggregations. Ann Hematol.

[44] Asch-Kendrick R, Cimino-Mathews A (2016). The role of GATA3 in breast carcinomas: a review. Hum Pathol.

[45] Li Y, Ishiguro H, Kawahara T, Miyamoto Y, Izumi K, Miyamoto H (2014). GATA3 in the urinary bladder: suppression of neoplastic transformation and down-regulation by androgens. Am J Cancer Res.

[46] Du F, Yuan P, Wang T, Zhao J, Zhao Z, Luo Y (2015). The significance and therapeutic potential of GATA3 expression and mutation in breast Cancer: a systematic review. Med Res Rev.

[47] Howitt BE, Emori MM, Drapkin R, Gaspar C, Barletta JA, Nucci MR (2015). GATA3 is a sensitive and specific marker of benign and malignant mesonephric lesions in the lower female genital tract. Am J Surg Pathol.

[48] Fu B, Guo M, Wang S, Campagna D, Luo M, Herman JG (2007). Evaluation of GATA-4 and GATA-5 methylation profiles in human pancreatic cancers indicate promoter methylation patterns distinct from other human tumor types. Cancer Biol Ther.

[49] Aronson BE, Stapleton KA, Krasinski SD (2014). Role of GATA factors in development, differentiation, and homeostasis of the small intestinal epithelium. Am J Physiol Gastrointest Liver Physiol.

[50] Wang H, Liu Z, Li J, Zhao X, Wang Z, Xu H (2012). DeltaNp63alpha mediates proliferation and apoptosis in human gastric cancer cells by the regulation of GATA-6. Neoplasma..

[51] Castro IC, Breiling A, Luetkenhaus K, Ceteci F, Hausmann S, Kress S (2013). MYC-induced epigenetic activation of GATA4 in lung adenocarcinoma. Molecular cancer research : MCR.

[52] Zois E, Vollstadt-Klein S, Hoffmann S, Reinhard I, Bach P, Charlet K (2016). GATA4 variant interaction with brain limbic structure and relapse risk: a voxel-based morphometry study. European Neuropsychopharmacology : the journal of the European College of Neuropsychopharmacology.

