# Supplementary figures and images for: Distinct expression and prognostic values of GATA transcription factor family in human ovarian cancer
Source: J Ovarian Res. 2022 Apr 29;15:49. doi: 10.1186/s13048-022-00974-6 (PMC9052646; doi:10.1186/s13048-022-00974-6)

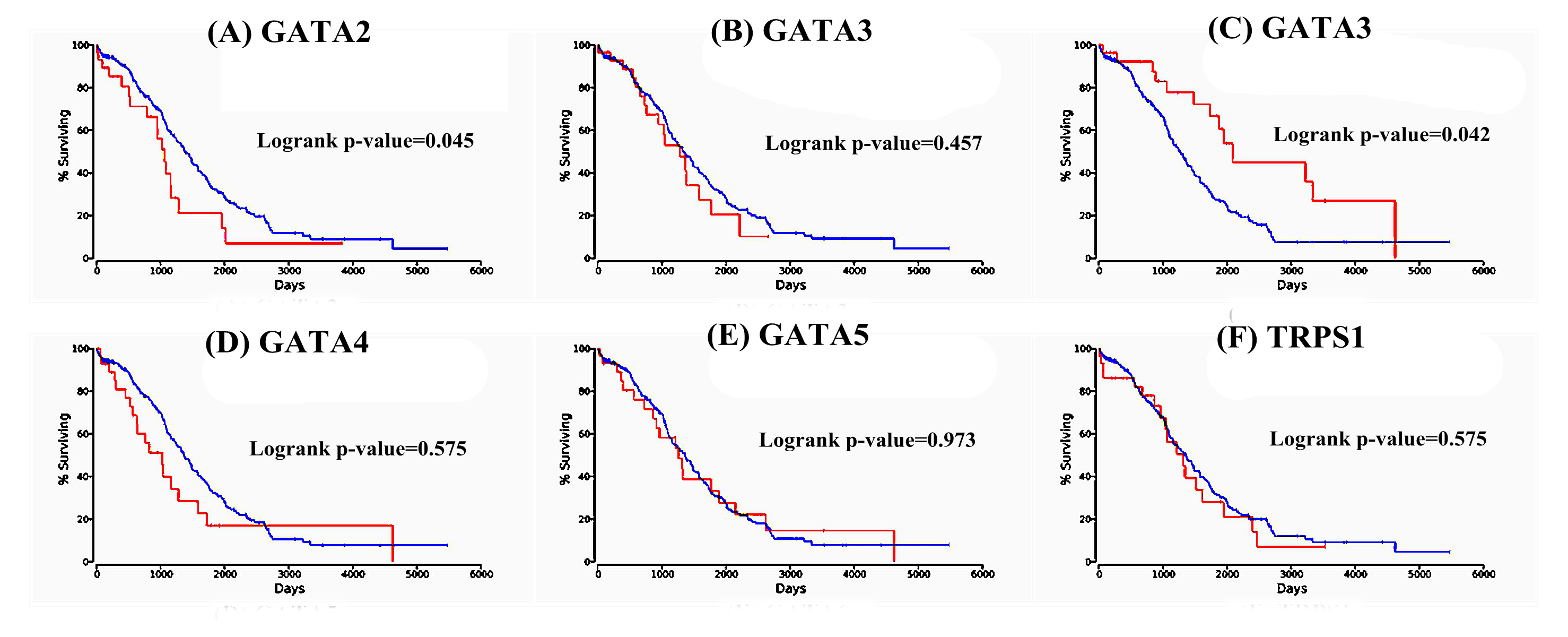

Supplement: Supplementary file 1 — Additional file 1: Fig. S1. The prognostic value of mRNA level of GATA family members in OC patients (OncoLnc online tool). There is no information in the OncoLnc database that correlates GATA1 expression with ovarian cancer prognosis. (A) GATA2 (Logrank p = 0.045), (B)GATA3 (Logrank p-value = 0.467), (C)GATA4(Logrank p-value = 0.042), (D)GATA5(Logrank p-value = 0.575), (E)GATA6 (Logrank p-value = 0.973), (F)TRPS1(Logrank p-value = 0.575) are plotted for all patients (n = 273) [file 13048_2022_974_MOESM1_ESM.tiff]
